# Immune Response and Molecular Mechanisms of Cardiovascular Adverse Effects of Spike Proteins from SARS-CoV-2 and mRNA Vaccines

**DOI:** 10.3390/biomedicines11020451

**Published:** 2023-02-03

**Authors:** Paolo Bellavite, Alessandra Ferraresi, Ciro Isidoro

**Affiliations:** 1 Independent Researcher, 37134 Verona, Italy; 2 Laboratory of Molecular Pathology, Department of Health Sciences, Università del Piemonte Orientale, 28100 Novara, Italy

**Keywords:** COVID-19 (Corona Virus Disease 2019), SARS-CoV-2 (severe acute respiratory syndrome coronavirus responsible for the COVID-19 disease), Spike, vaccine, immune response, thrombosis, myocarditis, inflammation, renin-angiotensin system, adversomics

## Abstract

The SARS-CoV-2 (severe acute respiratory syndrome coronavirus responsible for the COVID-19 disease) uses the Spike proteins of its envelope for infecting target cells expressing on the membrane the angiotensin converting enzyme 2 (ACE2) enzyme that acts as a receptor. To control the pandemic, genetically engineered vaccines have been designed for inducing neutralizing antibodies against the Spike proteins. These vaccines do not act like traditional protein-based vaccines, as they deliver the message in the form of mRNA or DNA to host cells that then produce and expose the Spike protein on the membrane (from which it can be shed in soluble form) to alert the immune system. Mass vaccination has brought to light various adverse effects associated with these genetically based vaccines, mainly affecting the circulatory and cardiovascular system. ACE2 is present as membrane-bound on several cell types, including the mucosa of the upper respiratory and of the gastrointestinal tracts, the endothelium, the platelets, and in soluble form in the plasma. The ACE2 enzyme converts the vasoconstrictor angiotensin II into peptides with vasodilator properties. Here we review the pathways for immunization and the molecular mechanisms through which the Spike protein, either from SARS-CoV-2 or encoded by the mRNA-based vaccines, interferes with the Renin-Angiotensin-System governed by ACE2, thus altering the homeostasis of the circulation and of the cardiovascular system. Understanding the molecular interactions of the Spike protein with ACE2 and the consequent impact on cardiovascular system homeostasis will direct the diagnosis and therapy of the vaccine-related adverse effects and provide information for development of a personalized vaccination that considers pathophysiological conditions predisposing to such adverse events.

## 1. Introduction

In December 2019, an outbreak of lung infections causing a respiratory distress disease with high lethality (at least in the first waves) emerged first in China and soon after spread worldwide, mainly through the European and American continents. The pathology features resembled the previously described SARS (severe acute respiratory syndrome) and was rapidly found to be caused by a novel beta coronavirus then named SARS-CoV-2 (severe acute respiratory syndrome coronavirus responsible for the COVID-19 disease) [1]. Due to the severity of disease, the lack of specific antivirals, and the purported pressure on health care systems (essentially requiring hospitalization in intensive care units), vaccination was considered the most promising and appropriate solution.

**Figure 1:**
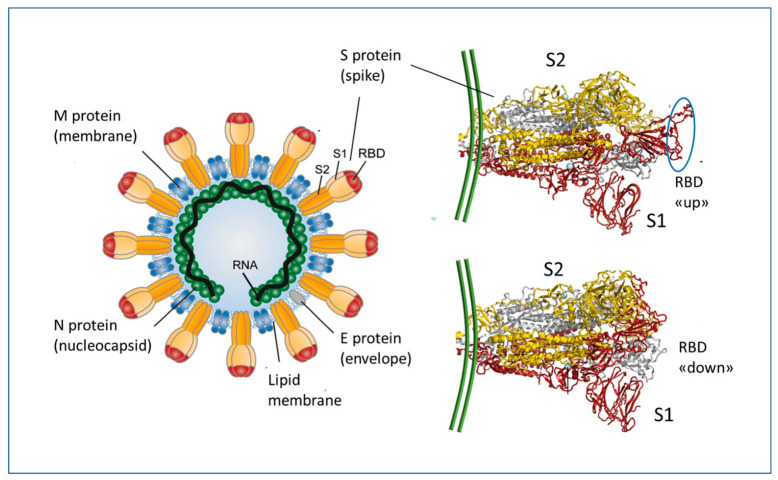
Structural organization of the SARS-CoV-2 (severe acute respiratory syndrome coronavirus responsible for the COVID-19 disease) virus and the Spike protein S (right). In the closed state, the receptor binding domain (RBD) is in the inactive (down) conformation and in the open state it is in the ‘up’ conformation, which can interact with the human ACE2 receptor. The interaction site is indicated by an ellipse in the upper right panel. Adapted from [7] Copyright 2020 Copyright Franz X. Heinz.

SARS-CoV-2, like other coronaviruses, uses the envelope Spike (S) glycoprotein for attaching to the cell through its binding to the protein angiotensin converting enzyme 2 (ACE2), exposed on the membrane of several cell types and thus acting as the virus receptor in the upper and lower respiratory tract, mouth, and intestinal mucosa [2,3,4,5] (Figure 1). The Spike protein is composed of two non-covalently bound subunits (S1 and S2) that arise from the furin-mediated cleavage of the S protein at the TGN (trans-Golgi network) during the virus transit [6]. The Spike proteins then assemble as trimers on the virus envelope, thus giving the crown-like aspect. It is to be noted that SARS-CoV-2-infected cells may express at the membrane some Spike proteins that have not been assembled into the virion, and from them the S1 could be released in soluble form [6]. The Spike binds to the ACE2 receptor via a part of the molecule called the RBD (receptor binding domain) in the S1 subunit, which in the prefusion state can assume the UP or DOWN configuration with the RBM (receptor binding motif), respectively accessible or not, for binding to the ACE2 [6] (Figure 1).

After interaction with the receptor, different variants of the virus can behave differently, in terms of infectivity and virulence, possibly due to different entry mechanisms. In fact, early variants preferred to use the entry mechanism involving the serine protease TMPRSS2, while not exploiting the endosomal mechanism through cathepsins; conversely, Omicron mainly uses the endosomal route with involvement of cathepsins and calpain [8,9]. How much these differences affect the effectiveness of vaccines is a matter of debate [10].

Based on this knowledge, scientists focused on the Spike protein as the best antigen candidate for immunization. Facing the urgency posed by the pandemic, gene engineering and transfection technologies were employed in the US, UK, and Europe, that allowed the rapid development and large-scale production of the vaccines as of December 2020 [11]. These vaccines were developed in a few months, which seems surprisingly quickly, thanks to the fact that the technology for mRNA transfer in vitro and in animals had been known for decades [12]. Thereafter, trials for assessing the efficacy and safety were run in parallel, for a relatively short period, which led to the vaccine’s emergency approval in a few months. Although they have formally been granted a marketing authorization, there is the need to provide further evidence of their efficacy and safety, based on the Phase 3 experimental studies and the Phase 4 observational studies that are still ongoing.

Several types of anti-COVID-19 vaccines have been made available and employed worldwide [13,14]. The Pfizer-BioNTech vaccine (BNT162b2, Comirnaty) and the Moderna vaccine (mRNA-1273, Spikevax), both using a lipid nanoparticle (LNP) platform for delivering the genetic information (mRNA) to instruct the synthesis of the Spike protein, were among the first vaccines to be approved for emergency use in December 2020 and are currently still the highest deployed types in the US and Europe. However, concerns have been raised regarding their efficacy to prevent virus transmissibility [15,16,17,18,19] and their safety [20,21,22,23,24].

Whether these vaccines fulfil the definition of “vaccine” or should instead be regarded as pro-pharmacologic drugs is a matter of debate [24]. However, for the sake of practicality, we shall not discuss here the name that better suits these immunostimulatory gene-based pro-drugs falling in the category of immunological-genetic product and will rather focus on their mechanisms of action. Here we will discuss how the mRNA-based vaccine elicits the immune response along with serious side effects on the cardiovascular system, whose severity depends on the distribution in the body of the Spike protein and the extent of the immune response elicited by the vaccine. 

Before entering the market and being authorized for large population immunization, vaccines should undergo extensive scrutiny to ensure not only their efficacy in preventing the infection or in reducing the extent of the manifestations of the disease caused by the infectious agent, but also and most importantly, their safety. This aspect is crucial, as vaccines are supposed to be administered to healthy people. Safety profiling of the vaccine becomes pivotal, especially when considering the need for frequent boosting because of immunity waning in only a few months [25,26]. In this respect, literature data report on a variety of serious adverse effects associated with COVID-19 mRNA vaccination [23]. These include myocarditis, pericarditis, hypertensive crisis, and other serious cardiovascular events [27,28,29,30,31], as well as neurological [32,33], dermatological [34], and autoimmune [35,36,37] reactions, among others.

Monitoring the potential adverse effects following immunization (AEFI), which could be coincidental and unrelated to the vaccine or could be a direct consequence of the vaccination, is fundamental to assessing the benefit/risk ratio [35,38,39,40,41]. Adverse events reported by the patients or the healthcare giver are collected in the database VAERS (Vaccine Adverse Event Reporting System) for US consumers (https://vaers.hhs.gov/ access date 24 July 2022) and the equivalent database Eudravigilance in Europe (https://www.ema.europa.eu/en/human-regulatory/research-development/pharmacovigilance/eudravigilance access date 24 July 2022), or AIFA in Italy (https://www.aifa.gov.it/farmacovigilanza-vaccini-covid-19 access date 24 July 2022).

The problem of the benefit/risk ratio of anti-COVID-19 vaccines is extremely complex for several reasons, including: (a) The disease severity is very different depending on age, gender and general health condition of the person. (b) The efficacy of vaccines wanes over time and changes according to the variants. (c) Pharmacovigilance data are obtained mainly through passive detection systems that are inadequate. The argument as to whether the risks of vaccination may in some circumstances outweigh the benefits of defence against disease is not within the scope of this paper, which focuses instead on the molecular mechanisms of adverse events following vaccination. Although the pathology associated with SARS-CoV-2 infection, especially with the variants prior to Omicron, was more intense than the pathology induced by the vaccine, the latter should not be neglected. Improving scientific knowledge of AEFI, even if in agreement with the hypothesis that serious ones are rare, means a lot in improving the general effectiveness of the vaccine prevention system. 

Basic sciences such as immunopathology, cell pathology, and the pathophysiology of the cardiovascular system may help to understand if and how such heart-related adverse events can indeed be mechanistically linked to the mRNA vaccination. Indeed, heart-related adverse events have been reported with anomalous high frequency, particularly in the cases of BNT162b2 (Pfizer-BioNTech) and mRNA-1273 (Moderna) mRNA vaccines. Instead, in the case of vaccines based on recombinant, replication-incompetent human adenovirus vectors, few case reports of myocarditis are present in literature [42,43,44]. Beyond the differences in technological platforms, it should be considered that mRNA vaccines have had a much wider diffusion and, moreover, require repeated administrations. Starting from the scientific theories explaining how anti-COVID-19 mRNA vaccines work, this paper focuses on the cellular, immunological, and pathophysiological mechanisms that could underlie the peculiar reactions in literature reported for the Spike protein, which is the main infectivity system of the virus and at the same time the main product against which vaccines intend to trigger the immune response. The present study contributes to further understanding of the potential toxic side effects, for a comprehensive assessment of the safety profile of these vaccines, which is instrumental to informing public health policy and to the prevention and/or cure of unwanted side effects.

## 2. Essentials of mRNA Vaccines Design and Functioning

Although various mechanisms of infectivity have been described [45,46,47], entry into cells by SARS-CoV-2 relies mainly on the interaction of the envelope Spike protein with cellular ACE2. Thus, blocking this interaction with an antibody seemed a good strategy. This prompted the vaccine production industry to design a genetically engineered vaccine capable of inducing in the host the production of neutralizing antibodies against the Spike protein, particularly toward the interacting region called the RBD. Indeed, the immunizing antigen is produced within the hosting cell once the nanoparticle load of mRNA is injected. Thus, for the vaccine to trigger the immune response, i.e., to elicit the biological (immunological) effect, the mRNA must be translated into the protein that, in turn, must interact with the immune system. 

The conception and rapid production of these new vaccines against SARS-CoV-2 followed within a few months after the Chinese authorities disclosed the sequence of the virus isolated in Wuhan. Western pharmaceutical companies rushed to use this sequence, and in particular the RNA “message” encoding the Spike protein, using a technology that was already available [12,48] yet never exploited on a large scale for human use. Seneff et al. [49] carefully and extensively analysed many of the critical points related to the engineered mRNA vaccines. Here we briefly report on those aspects regarding the immunogenicity of the exogenous vaccine mRNA, its entry into the cells, and its stability.

The vaccine mRNA was engineered to increase its stability, to escape cellular degradation, and to ensure the production of the Spike protein with the RBD accessible for inducing neutralizing antibodies [13,14]. It is noteworthy that the mRNA vaccine sequence maintains the furin cleavage site (a stretch of the four basic amino acids Arg- Arg- Ala- Arg at the S1–S2 junction) as in the viral sequence, and this has implications for the generation of the soluble S1 peptide [14,22].

The original sequence of the protein was slightly modified (i.e., K986 and V987 in the S2 subunit were substituted by two prolines) to direct the synthesis of the protein in a stabilized “pre-fusion” (open) conformation, like that interacting with the ACE2 cell receptors and to which neutralizing antibodies are supposed to react [13,14]. Other modifications are briefly described below. To allow entrance into the cells, the mRNA is encapsulated in lipid nanoparticles (LNPs) containing cholesterol and phospholipids associated with modified polyethylene glycol to avoid its degradation [50]. Viral RNA is recognized by the human cells as foreign, and this triggers defence reactions that impair its translation into proteins, while directing its degradation [51,52]. Replacing uridines with pseudouridines or (even better) with methyl-pseudouridine, overcomes the recognition as a foreign mRNA by the Toll-Like Receptors (TLR) and the subsequent activation of IFN type I [53]. To stabilize the mRNA and thus improve its translation, anti-COVID-19 mRNA vaccines have this characteristic [54]. To further stabilize the mRNA and increase the S protein production, a long poly(A) tail [55] and the 3′ UTR from human globin [56] were added to the mRNA molecule. A leader sequence, for translation in endoplasmic reticulum associated ribosomes, was added to ensure the insertion of the Spike protein into the plasma membrane. Notably, mRNAs vaccines are enriched in GC content: 53% in BNT162b2 and 61% in mRNA-1273 compared to 36% in native SARS-CoV-2 mRNA [57], and this also contributes to increasing the protein production [58]. Taken together, the vaccine mRNAs driving the Spike protein synthesis have been engineered in a manner that challenges the cellular stress response for the recognition of exogenous nucleic acids and proteins, and this is likely to impact the distribution of the mRNAs coding for the Spike protein and of the protein itself, which may then explain the biological and pathophysiological effects in organs distant from the site of injection. Indeed, the true biodistribution and the half-life of the vaccine mRNA in humans are currently unknown. Normally, mRNA is very fragile and is quickly degraded (within a few days). It was initially thought that vaccine mRNA would remain localized in the site of injection and be degraded within a few days, as is normal mRNA. However, real-world observations contradict this prediction. The S-protein has been detected in the plasma of mRNA-1273 COVID-19 vaccinees at 15 days following injection [59]. Both mRNA and S protein have been found in axillary lymph nodes after 60 days [60]. Very recently, Spike-mRNA has been detected in the blood of vaccinated individuals 15 and up to 28 days after COVID-19 vaccination [61,62]. Thus, it is likely that mRNA-LNPs remain in circulation for extended periods of time, retaining their ability to induce S protein expression in encountered cells. Updated bivalent mRNA vaccines that include the coding sequence for the Omicron BA.4/BA.5 variant were made available in September 2022, and studies on their efficacy and safety are still ongoing. Based on two pre-print studies, not yet peer-reviewed, the bivalent mRNA vaccine shows modest protection [63] and a higher rate of adverse events compared to the monovalent mRNA vaccine [64].

## 3. The Immune Response to the SARS-CoV-2 and to the mRNA Vaccines

The exact mechanism of stimulation of the immune system by the Spike protein-encoding vaccines is still hypothetical, and several versions exist. The first problem concerns the interaction of the injected product with the host.

### 3.1. The Importance of the Route of Entry 

Normally, pathogens enter the body via different routes, namely the oral and gastrointestinal mucosa, the nasal mucosa, the urogenital mucosa, and the skin. Each of these routes is characterized by a peculiar local microenvironment (stromal cells, tissue-specific factors, and commensal microbiota) which heavily influences the type and extent of the innate and specific immune response. When an infectious agent, a toxin, or a foreign antigenic molecule enters the body tissues or the blood, the immune system mounts a robust proinflammatory response, involving first the innate (non-antigen specific) immune system and, if required (depending on the type of antigen, route of entry and its persistence), the adaptive antigen-specific immune system. 

With evolution, the immune system has become more and more compartmentalized (cutaneous immune system, mucosal immune system, and systemic immune system) to improve its response and to reduce the risk of a dysregulated and disproportionate reaction. At the same time, the cells of the immune system can travel between the compartments and be influenced by the different local environments. The compartmentalized immune tissues communicate with each other to alert the system to the presence of the foreign potentially harmful “enemy” via the release of exosomes containing informative molecules (cytokines, microRNAs, PAMPs (Pathogen Associated Molecular Patterns), DAMPs (Damage Associated Molecular Patterns) from APCs and phagocytic cells [65]. Notably, circulating exosomes with inserted on the membrane the Spike protein have been detected in vaccinated individuals, and it is assumed that such exosomes are internalized by the APC, thus adding another route of immune sensitization [66]. 

The anatomical compartment determines the characteristics (differentiation status, phenotype, function, duration, turnover rate, homing capacity and regulatory mechanisms) of the immune cells. The threshold for activating the immune system is different in each organ and correlates inversely with its relative sterility [67,68]. To obtain effective and long-lasting protection at the site of entry, the pathogen must have direct contact with and be processed by the local tissue and compartmentalized immune system [67].

As with other respiratory viruses, in the case of SARS-CoV-2 infection, the early phase of humoral response is mediated by IgA antibodies that show greater neutralizing activity than IgG [69]. Upon viral infection, plasmablasts with homing receptors for mucosal sites and with intracellular IgA increase in the blood [69]. The fact that the level of secretory IgA specific for the Spike RBD in saliva was higher than that in the blood of the same subject 49 days after the onset of symptoms is indicative of the persistence of IgA in the oral mucosa [69]. 

The dimeric form of IgA, found in all secretions of both respiratory and intestinal mucosa, against SARS-CoV-2 is more potent than the monomeric IgA [70,71]. Salivary IgA specifically for the Spike protein are significantly lower in anti-COVID-19 mRNA vaccinees than in COVID-19 convalescent controls [72]. In fact, the current mRNA vaccines, though able to prevent/attenuate the most serious consequences of the disease, do not trigger the mucosal IgA response [73], even after the booster [74], and do not prevent the colonization of the virus in the mucous membranes [16]. The pattern of the cytokine response is also of paramount importance. The immune response to the virus and to the mRNA vaccines differ in that the former is characterized by strong induction of interferon and circulating effector B and T lymphocytes, whereas the latter is essentially restricted to circulating memory cells [75].

### 3.2. Immunization Pathways of the SARS-CoV-2 and mRNA Vaccines

mRNA COVID-19 vaccines are meant to induce B lymphocytes capable of producing antibodies against the (viral) S protein for preventing SARS-CoV-2 entry into the cells as well as T lymphocytes capable of killing the virus-infected cells (in the lung, kidney, etc.) expressing the S antigen on the membrane. However, the pathway for eliciting the immune response to the S protein coded by mRNA vaccines presents many peculiarities that need to be elucidated. 

**Figure 2:**
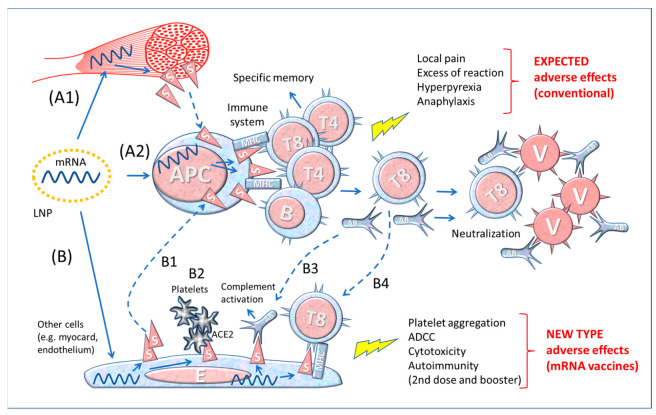
Diagram of the theory of functioning of anti-COVID-19 mRNA vaccines. A1–2 The simplistic theory representing the production and presentation of Spike protein by APCs to lymphocytes; A1: production of Spike by local muscle cells and release (by shedding) of soluble S that would be captured and processed by APC for immune stimulation; A2: the LNPs transfect the mRNA into APCs, which then produce and present in the context of MHC Spike to immune cells. B. Theoretical consequences of the expression of Spike protein by cell types other than immune cells transfected by mRNA containing LNP. B1: S protein released by somatic cells stimulates the immune system via APC; B2: interaction of blood platelets with S protein on the membrane of endothelial cells; B3: specific antibodies bind to S protein on the membrane of somatic cells (myocardium, endothelium, etc.) and activate the Complement system (or antibody-dependent cytotoxicity; not shown) leading to cell death; B4: specific CD8+ T lymphocytes (T8) attack endothelial cells expressing S protein. Abbreviations and symbols: LNP: lipid nanoparticle; APC: antigen-presenting cell; MHC: Major Histocompatibility Complex; S: Spike; T and B: lymphocytes; V: Virus; E: Endothelial cell; AB: Antibody; ADCC: Antibody-dependent cell cytotoxicity; ACE2: angiotensin-converting enzyme 2. Solid line arrows: action, operation; dashed line arrows: moving, displacement.

A common erroneous idea in the theory backing such mRNA vaccines is considering the Spike protein as a simple “foreign antigen” capable of stimulating immune defences, as it occurs for conventional vaccines. Let us consider the documentation provided for the first registration of Moderna’s mRNA-1273 vaccine to the US Federal Drug Administration [76]. In the presentation illustrating the immunization process [77], we read that the LNP loaded with the Spike-encoding mRNA would fuse with the plasma membrane of and release the mRNA into antigen presenting cells (APC), which in turn would manufacture the Spike protein and present it on the membrane to CD4+ T helper cells, CD8+ T cytotoxic cells and B cells. According to this theory, the mRNA vaccine: (a) “provides instruction (Spike protein) directly to the immune system” and (b) “efficiently creates specific immune memory in a natural context (in situ)”. This “theoretical” pathway is illustrated in the upper part (A2) of Figure 2.

This model reproposes the essential steps (not considering the complexities of the MHC system, chemical mediators, and accessory cells, etc.) of the theory of immunization with traditional vaccines made with microbe derivative substances or with the whole microbe after it has been attenuated, inactivated, or killed. However, the mRNA of vaccines is injected into muscular cells which produce and expose on the membrane the Spike protein that eventually could be shed and then captured by the APCs (Figure 2, A1 and A2). 

Conventional immunological knowledge teaches that antigen-presenting cells (APCs, dendritic cells, macrophages, and B memory cells) “capture” extracellular potentially pathogenic particles (showing a pathogen-associated molecular pattern, PAMP) by means of a series of appropriate receptors. These antigenic particles are then internalised by endocytosis or phagocytosis (depending on the particle dimension and the cell type), “processed” (i.e., digested) in small peptides (approx. 30 amino acids) and eventually inserted into the MHC-II (major histocompatibility complex) cleft for informing Th (CD4+) lymphocytes. However, for COVID-19 mRNA vaccines the scenario might be not so straightforward, as we will discuss below. 

The theory of “conventional” vaccinology predicts that immunity is obtained by injecting the foreign “antigen”, inactivated so as not to cause any harm to the host, yet still able to stimulate a specific humoral and cellular immune reaction [78]. According to this view, the expected adverse events following immunization are transient pain and inflammation at the injection site and transitory systemic symptoms such as fever and malaise. Albeit rarely, serious adverse effects may occur after vaccination, for instance due to an allergy condition (anaphylaxis) or to immune dysregulation or autoimmunity mediated by antigens themselves or by the adjuvant (e.g., aluminum particles), or possibly because of inadvertent use of improperly inactivated or mutated microbe (e.g., Sabin polio), or genetic susceptibility [40,79,80,81,82]. 

In the specific case of mRNA-driven antigen delivered via LNP, the following peculiarities should be considered: 1. The LNPs may fuse with the membrane of any cell they encounter and therein release the payload [83]. This implies that the mRNA may direct the synthesis of the Spike protein not exclusively in muscle cells but also in APCs and other somatic cells. 2. The mRNA is provided with a leader sequence, which directs the synthesis of the Spike protein in endoplasmic reticulum-associated ribosomes. The membrane bound S protein would then travel through the Golgi complex (here it will be split into S1 and S2 by furin) and then be exposed on the plasma membrane via insertional exocytosis [14]. Transfected cells could free the S protein and/or its fragments following T cell killing, and S1 (which is non-covalently bound to S2) could be shed from the membrane [14,22]. Consistently, high levels of soluble Spike proteins are found in the circulation of vaccinees with myocarditis [84]. The soluble Spike can be subsequently endocytosed by APCs and B lymphocytes. The transfected cells may release exosomes expressing the S protein on the membrane, which also contribute to immunostimulation of APCs in distant organs [66]. 

Antigen processing follows two different routes depending on the cell type (immune or non-immune) and whether the antigen locates in the endosomal compartments or in the cytoplasm. In the former case (occurring for instance in APCs), the exogenous antigen internalized via endocytosis/phagocytosis is proteolyzed by the endosomal cathepsins and the fragments inserted into the cleft of the MHC class II antigen (HLA-II) to be exposed on the plasma membrane for informing the CD4+ T helper lymphocytes. In the case of the virus infection (e.g., SARS-CoV-2) of parenchymal cells, viral proteins (for instance the S protein) in the cytoplasm are proteolyzed by the ubiquitin-proteasome pathway and the (immunodominant) peptides translocated into the endoplasmic reticulum where they are inserted into the cleft of MHC class I and eventually exposed on the plasma membrane. This will inform CD8+ T cytotoxic lymphocyte that the cell has been infected and should be killed. B lymphocytes, on their side, are stimulated by soluble antigens recognized by membrane B-cell receptors (a complex containing IgD or IgM) to become plasma cells producing and secreting soluble antibodies. There is crosstalk of cytokines between APCs, Th, Tc and B lymphocytes to orchestrate the immune response. 

However, in the case of the COVID-19 vaccination with LNP loaded with the modified mRNA we face unpredicted outcomes, since the mRNA transfection could aspecifically occur in any cell, including APCs, endothelial cells, and parenchymal cells of distant organs, wherein the mRNA would then direct the persistent synthesis of the modified (stabilized in open conformation) S protein. The processing route of the S protein will determine the fate of the transfected cells. 

In case of LNP transfection of parenchymal cells (ideally only the muscle cells at the injection site), the exposure on the membrane of the S protein would predictably trigger the CD8+ T lymphocyte cytotoxicity, much like what would happen to virus-infected cells. Yet, at variance from natural infection with SARS-CoV-2, in the transfected cells the S protein may (in part) not be processed, and be exposed on the membrane not in the context of the MHC class I. This eventuality could deceive the immune cells, which could consider the protein as a self. 

To add complexity, we must consider that other cells, in addition to APCs, can be transfected by the mRNA containing LNPs, as represented in Figure 2, bottom drawing (B). These cells would produce Spike proteins, display them on the membrane (or release after cell death or shed the S1) and trigger the response of the immune system (B1 in Figure 2). Furthermore, the Spikes exposed on the membrane of endothelium can interact with the ACE2 receptors exposed on the platelet membrane, favouring their aggregation (B2 in Figure 2). When the Spike synthesis is induced by boosters, i.e., in immunized individuals, the risk is that the transfected cells become victims of the aggression by previously formed antibodies (B3 in Figure 2) or by cytotoxic T8 lymphocytes (B4 in Figure 2). If this is the case, the adverse events following repetitive immunizations may be worse and involve various organs in which the Spike localizes. 

Hence, the mRNA vaccine “theory” neglects the possibility that any cell producing the Spike protein and displaying it on its membrane (associated or not with MHC-I) will be attacked and destroyed by CD8+T cells. The severity of the consequences for the host following the vaccination will depend on the type and number of cells affected and the tissue where the reaction occurs. For example, myocarditis is considered an adverse reaction to mRNA vaccination [85,86]. The facts that this event is more frequent after the second dose and it occurs a few days after the inoculation [27], suggest an immune-mediated mechanism analogous to an auto-immune reaction. To conclude, the Spike protein acts in a peculiar way, not simply as an immunogen, but as a disease-causing agent. 

### 3.3. Differences between Contact with the Whole Virus and Vaccine-Derived Spike Protein

Contact with the whole virus comprehensively instructs the immune system and all its components, therefore in case a constituent of the virus changes because of gene mutations, the immune memory toward the conserved viral components can still trigger the immune response. Furthermore, the different fragments of the virus presented by APCs to the lymphocytes trigger a complex polyclonal immune response that effectively neutralizes the virus. 

The components of a virus shape the type of the innate and specific immune response. A pathogen contains proteins, lipids, carbohydrates, and nucleic acids that constitute the so-called PAMPs that bind to the PRRs (Pattern Recognition Receptors) present on the APCs. The interaction leads to the maturation of the APC and the initiation of the adaptive immune response with the priming and differentiation of the antigen specific T helper cells, T cytotoxic cells, and B cells. The PAMPs combination determines the type (innate and/or adaptive), the extent, and the duration of the immune response. The biological and immunological implications of Spike immunization in relation to the type of vaccine, adjuvant, and route of administration have been studied in animal models [87].

All viruses have a specific cellular tropism, meaning that they enter and infect only those cells expressing the suitable receptor on their membranes. In the case of SARS-CoV-2, the virus preferentially enters the cells expressing the receptor for Spike (i.e., ACE2). On the contrary, as outlined above, the mRNA vaccines delivered via LNP can in principle (and in practice) transfer the information for the synthesis of the S protein to any cell. 

Many things about the vaccination outcome we still do not know: 1. Is the amount of S protein synthesized upon vaccination comparable with that of a natural virus infection or is it higher by many orders of magnitude? 2. How long does the Spike synthesis last following administration of mRNA? 3. How long do vaccine-derived Spike proteins remain biologically active? 

It is difficult to calculate exactly the number of copies of the Spike protein that results from the administration of these vaccines, because the declared amount of mRNA is not consistent in all batches (the producer Pfizer admitted that only 30 to 70% of the mRNA in the vaccine is integer for effective translation) and because its intracellular stability may vary from cell to cell.

Thus, it is reasonable to expect a big difference in the biological effect and the immune response between the natural infection and the administration of mRNA vaccines.

## 4. LNP Biodistribution and Spike Detection 

In the dossier submitted for mRNA-1273 authorization to the FDA, the vaccine producer (Moderna) claimed that immune reaction to the Spike would occur “in situ”, i.e., at the point of injection [77]. However, the few biodistribution studies carried out [88] showed that in mice and rats challenged with LNP labelled with radioactive probe or luciferase the signal is detected in various tissues, with the injection site, the spleen and the liver being the most enriched ones [89]. The technical dossier presented for the registration of Pfizer anti-COVID-19 vaccine reports that within 48 h from injection, LNP redistributed mainly to the liver, adrenal glands, spleen, and ovaries. 

Subsequent studies have shown the presence of vaccine-derived Spike proteins in the blood [59,90]. Since receptors for Spike are ubiquitously expressed in a variety of tissues and organs, it is likely that this protein performs activities that clearly go beyond its intended function as simple “antigen” [91,92]. Studies in laboratory animals have shown that Spike proteins may also cross the blood-brain barrier, which may account for neurological symptoms of the disease as well as of the vaccine [93].

Furthermore, immunohistochemical staining of axillary lymph node biopsies shows that vaccine Spike proteins were still present up to 60 days after the second dose of mRNA vaccines [60]. These authors found the Spike protein also in plasma in the first few days after vaccination (mean concentration of 47 pg/mL), yet the measurement of the Spike in blood after boosts was affected by the presence of specific antibodies. Circulating exosomes containing the Spike protein were found on day 14 after vaccination, and they increased after the booster dose, lasting up to four months [66]. While it has been suggested that these vesicles expressing the Spike protein on the membrane have the function of stimulating the immune response, it is not known whether they may interact with cells expressing ACE2. Vaccine-derived mRNA and Spike protein have been detected in the germinal centre of secondary lymphoid tissues two months after vaccination, suggesting sustained induction of protein synthesis [60]. Recently, circulating Spike proteins were detected in the blood of subjects hospitalized for myocarditis after mRNA vaccination [84]. Remarkably, the concentration of Spike protein (mean 33.9 ± 22.4 pg/mL) was significantly higher in symptomatic vaccinees than in asymptomatic ones, and it was measurable until three weeks after vaccination [84].

The Spike protein was detected by immunohistochemistry in the vessel wall of the brain and heart of a 76-year-old patient deceased three weeks after receiving his third COVID-19 vaccination [94]. Since no nucleocapsid (N) protein was detected, the authors suggest that the pathology was caused by vaccination and not by SARS-CoV-2 virus infection.

It is to be stressed that free Spike proteins in the plasma have also been found in the course of the COVID-19 disease, which can explain some clinical and pathophysiological manifestations. Circulating free S1 (the extracellular subunit containing the RBD) protein was detected in a substantial amount in patients, particularly in seriously ill ones, and likely contributed to endothelial dysregulation and thrombosis [95]. The Spike protein was detected in platelets from COVID-19 patients’ thrombi, in the absence of SARS-CoV-2 RNA, suggesting its involvement in platelet activation and clot formation [96].

Another troubling study on the Pfizer vaccine presents evidence of the possible permanence of the message inside the cell in the form of DNA [97]. According to this study, the rapid entry of mRNA into human liver cells would be followed by “reverse transcription” to the DNA within a few hours [97]. Whether the DNA reverse transcribed from BNT162b2 mRNA is integrated into the cell genome has not been proved, yet the finding raises the concern that the integrity of genomic DNA could be affected, underscoring possible genotoxic side effects. Furthermore, if the mRNA message is retro-transcribed in DNA, which is more stable, the synthesis of the Spike proteins may persist for long time.

## 5. The “Active” Spike and the Renin-Angiotensin System

ACE2 is a transmembrane enzyme localized in many organs including lung, kidney, endothelial cells [98,99], platelets [46], mast cells [100,101], brain [102], testicles, prostate and uterus [103], digestive system cells such as oral mucosa, salivary glands, enterocytes, cholangiocytes of the liver and in adipose tissue [99]. The widespread distribution of ACE2 may explain the multi-organ damages caused by the Spike, either coded upon SARS-CoV-2 infection or following mRNA vaccination. 

In fact, apart from a few minor modifications made to stabilise the protein in the open conformation, the “wild” (i.e., viral) Spike and the “synthetic” (from the mRNA vaccine) Spike have the same biochemical features and, more importantly, the same pathological functions [22,92,104]. In other words, the vaccine-derived Spike proteins “mimic” the behaviour of the virus-derived homologs, and the pathology depends on the organs in which the Spikes are formed and distributed. 

**Figure 3:**
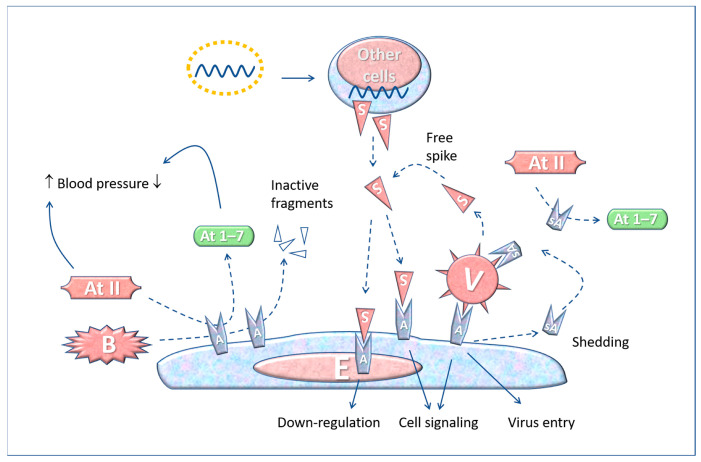
The interaction between SARS-CoV-2 and free Spike protein with ACE2 receptors on the membrane of an endothelial cell. The interaction favours the virus entering the cell (e.g., platelets, leucocytes, macrophages, endothelia) as well as the cell activation by the free Spike. ACE2 can convert angiotensin II (made from 8 amino acids) into an inactive form (1–7), and can inactivate bradykinin, a major mediator of acute inflammation. The Spike-ACE2 interaction can lead to platelet aggregation, inflammation and thrombosis, as described in the text. Abbreviations and symbols: E: Endothelial cell; V: Virus; S: Spike; A: ACE2; sA: soluble ACE2; ATII: angiotensin II; At 1–7: angiotensin 1–7; B: Bradykinin. Solid line arrows: action, operation, effect; dashed line arrows: moving, conversion.

The engagement of ACE2 receptor by the Spike protein, either from the virus or the vaccine, alters the equilibrium in the Renin-Angiotensin-System (RAS), and this has various consequences in the pathophysiology of the blood and cardiovascular system, as shown in Figure 3.

The cellular entry of SARS-CoV-2 through ACE2 has considerable consequences in the course of the COVID-19 disease [105], and when binding to the ACE2 receptors on platelets may cause thrombosis. In leukocytes, other receptors, besides ACE2, can be targeted by Spike proteins [106,107].

Given the biochemical similarity between the virus-derived and vaccine-derived Spikes, it is expected that the latter also affects the RAS with possible pathological consequences, especially on blood pressure and circulation [104,108,109,110,111,112,113].

**Figure 4:**
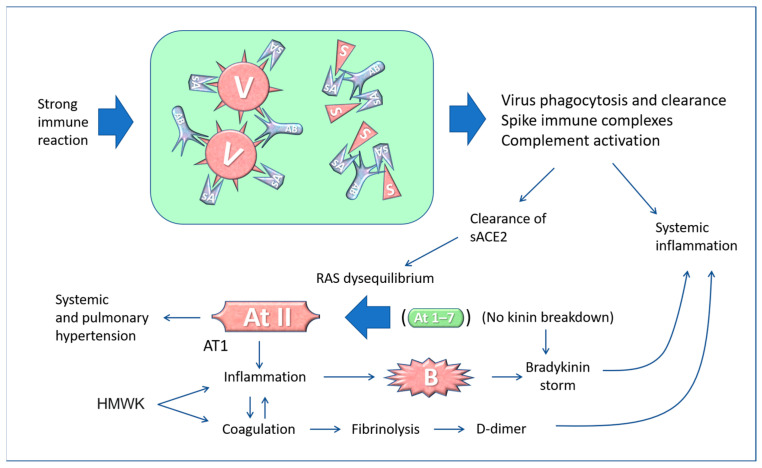
Pathological effects of COVID-19 and the vaccine on the renin-angiotensin system (the balance between angiotensin II and angiotensin 1–7 is illustrated here). ACE2 normally destroys angiotensin II, an 8-amino acid peptide that has a hypertensive action and causes water retention, and converts it into “angiotensin 1–7”, which has a hypotensive effect. Abbreviations and symbols: RAS: Renin-angiotensin system; E: Endothelial cell; V: Virus; S: Spike; A: ACE2; sA: soluble ACE2; ATII: Angiotensin II; At 1–7: Angiotensin 1–7; B: Bradykinin; Ab: Antibody; HMWK: High molecular weight kininogen; AT1: Angiotensin II receptor type 1.

Besides the membrane form of ACE2 (called “mACE2”), a soluble form called “sACE2” can be found free in the plasma. ACE2 shedding (and formation of sACE2) is a well-known process mediated by ADAM17 (TACE)-mediated proteolysis of the membrane-bound form [114]. When SARS-CoV-2 binds to the target cells, a certain amount of ACE2 molecules is released from the membranes by the action of proteolytic enzymes and pass into the plasma, where they can decrease angiotensin II level, thus leading to hypotension [113]. In COVID-19 patients, ACE2 shedding is exacerbated and the plasma level of sACE2 correlates with COVID-19 severity [115]. It is conceivable that a similar effect occurs in mRNA vaccinated individuals because of the soluble Spike (and the exosomes exposing membrane-bound Spike) (Figure 4). 

Furthermore, the Spike proteins enhance platelet aggregation, thus promoting thrombosis [116], and activate endothelial cells via ACE2, thus increasing leukocyte recruitment, adhesion, and complement activation [95].

The biggest problem arises when the sACE2-virus or the sACE2-Spike complexes are cleared by antibodies (formed a few days after the onset of the disease or after vaccination) or by phagocytic cells. This leads to reduced ACE2 activity and, consequently, an increased level of the hypertensive angiotensin II and alteration of the inflammation, coagulation, and hydroelectrolytic systems [117] (Figure 4). 

A similar scenario may happen after re-infection in vaccinated individuals. The binding of coronavirus Spike protein to the ACE2 receptor causes its internalisation [118], and this leads to a net decrease in ACE2 enzyme activity, which then results in an increase in angiotensin II and consequently in increased blood pressure and bradykinin accumulation. Also, the binding of Spike to membrane-bound ACE2 can cause pulmonary injury and vasoconstriction because of impaired conversion of Angiotensin II to Angiotensin 1–7 [113,119]. 

**Figure 5:**
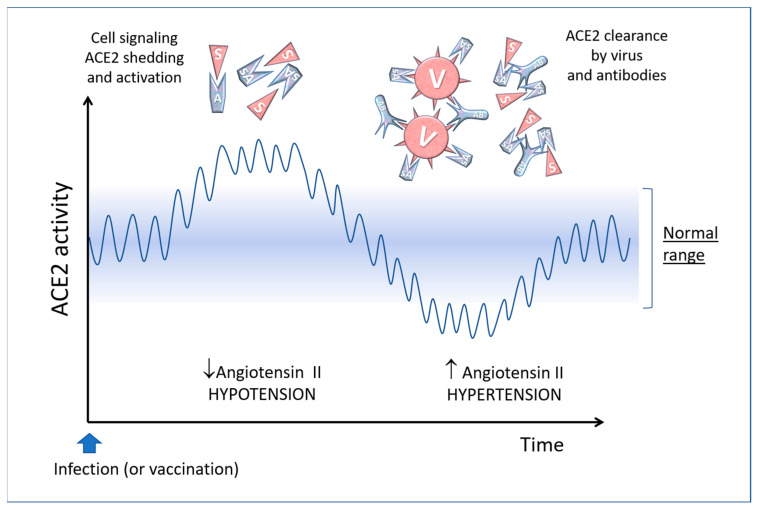
Conceptual diagram of possible imbalances in the renin-angiotensin system caused by the interaction of antibodies with SARS-CoV-2 or vaccine-derived Spike proteins. Adapted from [104] Copyright 2021 Copyright Paolo Bellavite.

Furthermore, imbalances arise in the kinin system up to the so-called “kinine storm” [120,121]. In fact, ACE2 also regulates the kinin system by eliminating bradykinin, which is responsible for inflammatory phenomena and exudates. It has been shown that key elements of the bradykinin, angiotensin and coagulation systems are co-expressed with ACE2 in lung alveolar cells, and this could explain how changes in membrane ACE2 caused by the virus determine the development of the most severe clinical forms of COVID-19 [122,123]. In fact, bradykinin-mediated inflammation contributes to life-threatening respiratory complications in COVID-19 [124], and this is one of the reasons for recommending an anti-inflammatory in the treatment of COVID-19 patients [125]. In addition, minimal Spike doses, added to human whole blood in vitro, induce the production of many types of cytokines, growth factors, chemokines and RANTES (regulated upon activation, normal T-cells expressed and secreted) [126]. As stated, the antibody-mediated clearance of the virus complexed with sACE2 causes a rapid decline of circulating ACE2. Similarly, the free Spike (and the Spike-containing exosome) from the mRNA vaccine can lead to an upheaval of the RAS that may cause an increase in blood pressure and hyperinflammatory reactions [92,104,113,127,128] (Figure 5).

Consistently, acute and significant elevation of blood pressure has been reported as an adverse reaction of anti-COVID-19 [127]. Hypertensive crisis can have serious and even tragic consequences, being an established risk factor for subarachnoid cerebral haemorrhage, increasing the risk by 2.6 times (as a comparison, smoking increases it by 3.1 times and alcohol abuse by 1.5 times) [129].

Compared to the influenza vaccine, COVID-19 mRNA vaccines have a much higher risk for hypertensive crisis (adjusted odds ratio 12.72, 95% CI 2.47–65.54) and supraventricular tachycardia (adjusted odds ratio 7.94, 95% CI 2.62–24.00) [30]. The risk is as much as 12 times higher with the anti-COVID-19 mRNA vaccine than with the anti-influenza vaccine. 

## 6. Molecular Mimicry and Anti-Idiotype Antibodies

The Spike protein presents some motifs common with human proteins, among which is a stretch of five amino acids (namely, TQLPP) with antigenic properties that are homologous with a sequence found in thrombopoietin, and the motif ELDKY that is shared with tropomyosin and with Protein Kinase cGMP-dependent type 1 (PRKG1), a kinase involved in platelet activation and calcium regulation [37,130]. 

Molecular mimicry is one of the mechanisms hypothesized to explain the development of autoimmune disease. An important concern is whether the mRNA vaccination for producing the Spike protein could determine a break in the tolerance and development of an autoimmune disease because of the molecular mimicry. The risk increases with frequent and close together administrations of the vaccine, that challenge the immunogenic versus tolerogenic state of the immune system. In this condition, proinflammatory cytokines may alter the control of immunoregulatory circuitry so that self-reactive T cells could become effective and trigger autoimmunity [131]. In addition, the “homologies” between the Spike protein and human proteins are much greater than for other viruses and bacteria, increasing the risk of developing autoimmune diseases.

**Figure 6:**
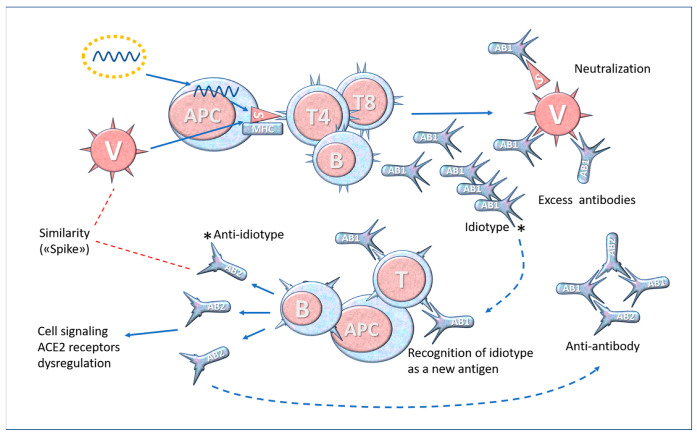
Simplified diagram of anti-idiotype antibody formation. A variable part of the antibody (Ab1) able to bind to the Spike is called idiotype. Since this part is a “new” protein within the repertoire of antigens known to the immune system, the latter produces antibodies (Ab2) in response to the idiotype, which can recognise and bind to it. These secondary antibodies, called “anti-idiotype”, represent in a certain way the internal image of the external antigen (Spike) and may share some of its biological properties. * Molecular complementarity of idiotypes.

The issue of interactions between the immune system and ACE2 (or other virus receptors) is further complicated when considering that the immune system is complex and dynamic. This is illustrated by the formation of “anti-idiotype” antibodies (see Figure 6).

The formation of anti-idiotype antibodies and lymphocytes is a possible explanation for the persistence of symptoms typical of COVID-19 even after the virus has been eliminated from the body. In accordance with Jerne’s well-established theory [132], these antibodies “look like” the critical part of the Spike. Thus, the anti-idiotype antibodies (Ab2 in Figure 6), which reflect the Spike epitope, may bind to ACE2 or similar structures and cause the pathophysiological reaction described above. Anti-idiotype antibodies against ACE2 were found in 81% of COVID-19 patients but not found in unaffected patients [133]. The authors hypothesized a role of these antibodies in explaining COVID-19-associated cardiovascular events. This phenomenon can occur with the SARS-CoV-2 infection as well as with anti-COVID-19 vaccines [134], explaining at least in part the persistence of adverse reactions in some individuals. It has also been suggested that anti-idiotype antibodies could bind to neuropilin-1, which is recognized by the Spike of the SARS-CoV-2 virus [135], and this could explain some neurological adverse effects such as peripheral neuropathy arising after vaccination with BNT162b2 [136].

## 7. The “Boost” and Trained Immunity

Even innate defence cells can develop immune memory characteristics, a process called trained immunity [137,138]. Many inflammatory insults can alter the functionality and reactivity of the innate immune system in the long run, and this could be relevant when the stimuli are reiterated, as in the case of repeated vaccinations.

Because of the rapid loss of protective efficacy induced by the current mRNA vaccines, multiple administrations were envisaged with the idea of giving the immune system a periodical “boost”. The consequence of these repeated booster doses over time, in the short, medium, and long term are unknown. Assessment of the safety of repeated booster doses stimulating the immune system should also consider the functioning of innate immunity.

**Figure 7:**
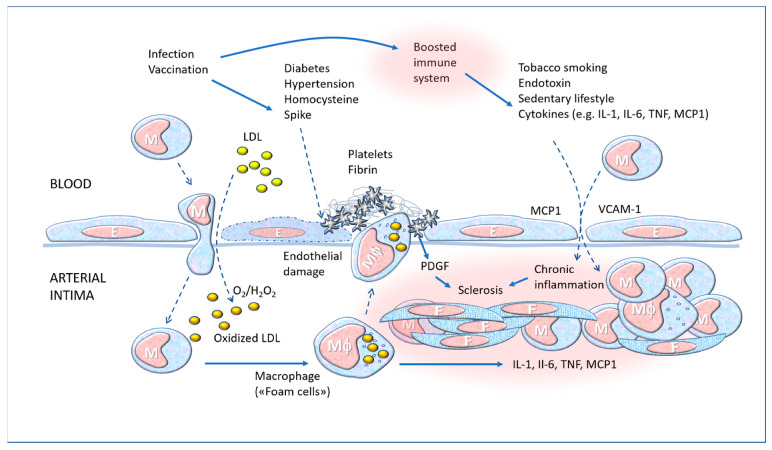
Essential mechanisms of pathogenesis of atherosclerosis, seen as a chronic inflammatory disease. M: Monocyte; Mϕ: Macrophage; E: Endothelial cell; F: Fibroblast; LDL: low-density lipoprotein; VCAM-1: vascular cell adhesion protein 1; PDGF: platelet-derived growth factor; TNF: tumour necrosis factor; IL: interleukin; MCP1: Monocyte Chemotactic Protein-1 (CCL2). Solid line arrows: action, operation; dashed line arrows: moving, displacement.

It should be emphasised that innate immunity has a long-term memory due to the “epigenetic” reprogramming of cellular chromatin, and the capacity for increased responsiveness remains when inflammation resolves [139]. In monocytes and macrophages, this epigenetic reprogramming was associated with increased cytokine production and a metabolic shift from oxidative phosphorylation to glycolysis [140]. This could be an advantage in terms of specific response, but the same trained immunity can become “maladaptive” in diseases characterised by chronic systemic inflammation, such as atherosclerosis and cancer [137,138,139,140]. In addition, certain non-immune cells such as endothelial cells and fibroblasts also display trained immune characteristics, and this has been seen also in relation to coronavirus infection [141]. In the long term, a possible outcome of COVID-19 as well as of repetitive boosters is the development or exacerbation of pre-existing atherosclerosis, given that this is a chronic inflammatory disease of the vascular wall also involving monocyte-macrophage phagocytic cells [142] (see Figure 7).

Thus, repeated administration of booster doses a few months apart could have positive and desirable effects if strengthening a specific immunity (antibody or T-cells), but it could have negative effects in stimulating “non-specific” reactive capacity based on the trained immunity of endothelial and macrophage cells. These cells are not only capable of stimulating the lymphocyte system (which is desirable in the context of a well-functioning system, except in the case of autoimmunity), but are involved also in multiple pathological processes characterised by chronic inflammation, such as cardiovascular diseases, diabetes, osteoarthritis, and others. 

Further studies and tests will confirm whether the repeated administration of vaccine stimuli in the long term will have a negative impact on the cardiovascular system. This poses the question of whether the risk of contracting the viral disease, which causes strong, acute reactions but leaves complete and lasting immunity [143], is comparable with the risk of side effects of vaccination, which having short-term protection requires repeated administrations (every 3–5 months) and thus could trigger or worsen chronic inflammatory pathologies.

## 8. Overview and Prospects

The mechanisms by which the free Spike protein may act in living systems are summarized in Table 1. 

In addition, other mechanisms that could contribute to the COVID-19 vaccine-associated cardiovascular disorders should be considered [169]. It has been hypothesized that COVID-19 vaccination could aggravate a pre-existing T-mediated heart-specific autoimmunity. Infiltration of CD3+ T lymphocytes has been reported in acute myocarditis following BNT162b2 mRNA COVID-19 vaccination [170]. A role for sex hormone on myocardial inflammation upon COVID-19 infection or mRNA vaccination also should be considered, given that testosterone and estrogen elicit opposite effects on T cell response. 

These mechanisms are not independent and can overlap and act synergistically. This opens a new chapter in vaccinology, perhaps unexpected for the inventors of vaccines themselves, which should be investigated in depth since the pathologies associated have an enormous impact on vaccine risk assessment. Moreover, knowledge of the mechanistic factors involved in vaccine damage could prepare for better diagnostics (e.g., D-dimer, histamine or tryptase measure, plasma cytokine patterns, accurate blood pressure measurement, troponin, genetic risk assessment, etc.) and therapeutic interventions. 

### 8.1. Causality Assessment

Immunization with the COVID-19 mRNA vaccines is particularly challenging for the immune system and has important reflections on the pathophysiology of the cardiovascular system because: 1. These are not traditional vaccines, but instead behave as immunomodulatory pro-drugs that are “metabolized” for producing the active antigen in an unpredicted amount, in unpredicted sites (tissue, cell type), and for unpredicted lengths of time. 2. The encoded Spike protein is not simply an antigen; instead, it is an active RAS modulator. 3. The encoded Spike protein may not reside on the membrane of the transfected cells, but instead can be released in a free form or bound to exosomes and travel to sites distant from the synthesis site. 

The above considerations are important when assessing the causation of any adverse event after vaccination involving the cardiovascular system, such as cardiac arrest, stroke, haemorrhage, and shock. The correlative nexus does not necessarily imply a causative nexus. In this regard, WHO has elaborated guidelines for the causality assessment of an adverse event following vaccination in which all “other possible causes” that could have led to the event should be considered [171,172]. The medical history and clinical examinations of the patient along with laboratory data help identify other diseases or congenital anomalies that could have caused the event. However, in addition to the presence of a temporal correlation and the absence of another “strong” cause not related to the possible effect of the vaccine, the plausibility of the explanation of the possible pathogenic effect of the vaccine is very important [40]. For instance, a sudden rise in blood pressure could be fatal in people with brain aneurysms, a problem aggravated by possible thrombocytopenia. For this reason, understanding the Spike protein’s mechanisms of action is essential, especially in the event of unforeseen and inexplicable effects based on knowledge accumulated with previous “conventional” vaccines.

As an example of the difficulties of a genuine correlation assessment, consider the results contained in the report on the 6-months BNT162b2 vaccine trial [173]. It states that during the study period, 15 participants died in the vaccine-treated group and 14 in the placebo group, and that none of these deaths were related to BNT162b2 according to the investigators. However, on closer inspection, Table S4 of that paper [173] shows that among the deaths in the vaccine-treated group, four were due to “cardiac arrest” and two to “atherosclerosis”, whereas in the placebo-treated group the deaths due to these two conditions were 1 and 0, respectively. The fact that vaccine-derived Spike proteins can have a dysregulatory influence on the RAS implies that, in the case of patients with cardiovascular and coagulation diseases, an interaction between the vaccine and the underlying condition is entirely plausible and should not be discarded. 

### 8.2. Diagnostic and Therapeutic Implications

Knowledge of the complexity and variety of reactions underlying the use of Spike protein-based vaccines suggests greater attention to the individualization of vaccine administration. The paradigm of mass vaccination, regardless of the individual assessment of the expected benefits and risks of immunization, may be understandable (if not acceptable) at the beginning of an emergency vaccination campaign, but now a careful and personalized approach is necessary as has been proposed for other vaccines [174,175].

Knowledge of the molecular interactions of the Spike protein and its impact on the homeostasis of the organism can help in bettering pre-vaccination diagnostic activity. For example, blood pressure, coagulation parameters, the presence of potentially interacting risk factors such as those mentioned above (Figure 7), and genetic susceptibility to inflammatory and autoimmune diseases should be carefully evaluated. As with traditional vaccines, it would be possible to develop a program for the systematic detection of adverse effects and associating them with immunogenetic and cardiovascular characteristics, to build a predictive map of the risks [176,177]. It would be important to evaluate the different cytokine patterns, which could determine the greater or lesser systemic reaction to vaccination, as was reported after smallpox immunization [178,179,180,181]. It has been clearly established that some genetic background characteristics, such as cytokines or ACE2 polymorphisms, can potentially explain the large interindividual variation of COVID-19 disease [182]. It is therefore plausible that the development of tests aimed at identifying specific ACE2 variants could be a strategy to also evaluate the risk of adverse reaction to vaccination. Additionally, awareness of the cardiovascular risk linked to adverse reactions to vaccines can trigger diagnostic suspicion in the case of vague and non-specific symptoms. For instance, troponin dosage is a valid marker of cardiac damage and could be informative even in the event of an autopsy, provided it is performed within 48 h of death [183].

Most importantly, therapies for the most serious adverse reactions must be based on the full understanding of the mechanism(s) involved. For example, if an imbalance in the RAS system is suspected, the use of angiotensin II or bradykinin inhibitors could be considered; if an implication of prevalent blood clotting is suspected by symptoms or by an increase in D-dimer, the use of platelet aggregation inhibitors or anticoagulants could be considered; if allergic or urticarial manifestations are observed (due to mast cell involvement, with possible observation of an increase in histamine or tryptase), the use of antihistamines could be considered; if the prevailing pathogenetic hypothesis focuses on autoimmunity in the case of severe neurological pathologies, the use of corticosteroids or immunosuppressants is indicated.

Given the similar pathogenic action of the SARS-CoV-2 and the vaccine mRNA encoded Spike protein, it seems plausible that molecules capable of blocking the virus binding to ACE2 receptors could likewise prevent or counteract the adverse events of vaccination. A variety of natural and synthetic molecules capable of binding to the RBD fragment of the Spike and to ACE2 have been identified [184,185,186,187,188,189,190,191]. Whether these molecules or others with immunomodulatory properties (anti-allergic or anti-cytokine drugs) are useful in the prevention or treatment of adverse reactions to vaccines should be assessed through appropriate randomized clinical trials. Finally, a rational approach would be to harness the omics technology for the design of more efficacious and safe vaccines as well as for understanding the mechanistic causes of the vaccine’s adverse effects for a better personalized assessment of the benefit/risk ratio of vaccination [40,174,175].

## Figures and Tables

**Table 1:** Molecular, cellular, and immunological mechanisms of pathogenic effects of free Spike protein. A synopsis of the studies reporting the possible clinical effects, and the underlying mechanisms, caused by the expression of the Spike protein either coded by SARS-CoV-2 or the mRNA vaccine. Mechanisms include the molecular interaction of the S protein with membrane-bound or soluble peptides (e.g., ACE2, sACE2, CD147, PAF, PF4, TLRs), the molecular mimicry, the induction of autoantibodies and of anti-idiotype antibodies, altered gene expression, alternative splicing and immune printing (for details, refer to the references). PAF, Platelet Activating Factor; PF4, Platelet Factor 4; TLR, Toll-like receptor.

Molecular Mechanisms	Pathogenic Mechanisms	Possible Clinical Effects	Refs.
Spike-ACE2	Platelet hyperreactivity and aggregation	Thrombosis	[116,144]
Spike-ACE2	Human endothelial cell activation and pro-inflammatory phenotype	Inflammation, thrombosis	[95]
Spike-ACE2	Inhibition of hematopoietic stem cells differentiation	Immunosuppression	[145]
Spike (S1)-ACE2	Intratracheal S1 subunit of Spike protein in hACE2 transgenic mice that overexpress human ACE2	Lung vascular permeability and lung injury	[146]
Spike-ACE2	Mast cell activation	Lung inflammation and injury	[144]
Spike-ACE2	Oxidative stress in pericytes, activation of nuclear factor-kappa-B signaling pathways	Encephalitis	[147]
Spike-ACE2	Down-regulation of endothelial ACE2 and e-NOS, mitochondrial damage	Interstitial pneumonia	[148]
Spike-ACE2	Decrease of type I interferons in lung primary cells	Severity of pneumonia	[149]
Spike (S1)-ACE2	S1 subunit co-localized with caspase-3, ACE2, IL6, TNFα, and C5b-9 (mice brain endothelia)	Inflammation and neuropathology	[150]
Spike (S1)-ACE2	S1 subunit elicits MEK/ERK pathway cell signaling in lung vascular cells.	Pulmonary vascular wall thickening, pulmonary hypertension	[92]
Spike-ACE2	Decrease of taste buds of rat circumvallate papillae	Taste disorders	[151]
Spike-ACE2	Loss of integrity of the human brain-blood barrier	Pro-inflammatory response on brain	[93,152,153,154,155]
Spike (S1)-ACE2	Loss of integrity of human pulmonary arterial endothelial cells	Pro-inflammatory response on lung	[156]
Spike-sACE2-antibodies	Soluble ACE2 internalization and clearance	Hypertensive crisis, inflammation, bradykinin storm	[104,113]
Spike-CD147	Cell signaling in human cardiac pericytes, secretion of cytokines, apoptosis	Cardiac microvascular damage	[157]
Spike-CD147	Cell signaling in human platelets	Thrombosis, inflammation	[158]
Spike-PAF	Augmentation of in vitro PAF-induced platelet aggregation and stimulation of U-937 (myeloid lineage) PAF production	Inflammatory syndromes, long COVID-19	[159]
Molecular mimicry	Cross-reaction of anti-Spike antibodies with pericardium	Pericarditis	[130,160]
Molecular mimicry	Cross-reaction of anti-Spike antibodies with thrombopoietin and with tropomyosin	Thrombocytopenia, myocarditis	[37,161]
Spike-autoantibody	Thyroid inflammation	Subacute thyroiditis	[162]
Spike-PF4 interaction	Generation of anti-PF4 antibodies and binding to platelet ACE2	Thrombosis with thrombocytopenia	[163]
Anti-PF4 antibodies	Platelet activation and aggregation	Thrombosis with thrombocytopenia	[164,165]
Anti-idiotype	Anti-idiotype (Ab2) would bind to ACE2 and/or to neuropilin-1	COVID-19-like symptoms	[134,136]
Gene expression	Decrease of ACE2 and increase of ACE	Inflammation, myocarditis	[166]
Spike-TLR4	The S protein triggers TLRs and induces inflammatory cytokines	Worsening of inflammatory reactions	[167]
Immune imprinting	Vaccine immune memory against S protein of the original variant inhibits the response to new epitopes of SARS-CoV-2	Increased susceptibility to COVID-19 variants	[168]

## Data Availability

Not applicable.

## Abbreviations

Adverse effects following immunization, AEFI; angiotensin converting enzyme 2, ACE2; angiotensin, At; antibody, Ab; antibody-dependent cell cytotoxicity, ADCC; antigen presenting cells, APCs; coronavirus disease 2019, COVID-19; coronavirus, CoV; damage-associated molecular patterns, DAMPs; envelope, E protein; high molecular weight kininogen, HMWK; human leukocyte antigen, HLA; immunoglobulin, Ig; interferon, IFN; interleukin, IL; lipid nanoparticle, LNP; low-density lipoprotein, LDL; major histocompatibility complex, MHC; membrane, M protein; monocyte chemotactic protein-1, MCP1; nucleocapsid, N protein; pathogen-associated molecular patterns, PAMPs; pattern recognition receptors, PRRs; platelet activating factor, PAF; platelet factor 4, PF4; platelet-derived growth factor, PDGF; receptor binding domain, RBD; receptor binding motif, RBM; regulated upon activation, normal T-cells expressed and secreted; RANTES; renin-angiotensin system, RAS; severe acute respiratory syndrome, SARS; soluble ACE2, sACE2; spike, S protein; Toll-like receptors, TLR; trans-Golgi network, TGN; tumour necrosis factor, TNF; vascular cell adhesion protein 1, VCAM-1; world health organization, WHO.
